# Comparison Between Chronological and Bone Age at Menarche in Girls with Laron Syndrome

**DOI:** 10.3390/children13070861

**Published:** 2026-06-29

**Authors:** Avivah Silbergeld, Zvi Laron

**Affiliations:** 1 Endocrinology and Diabetes Research Unit, Schneider Children’s Medical Center, Petah Tikva 4920235, Israel; avivya888@gmail.com; 2 Gray Faculty of Medical and Health Sciences, Tel Aviv University, Tel Aviv 69978, Israel

**Keywords:** age at menarche, Laron Syndrome, IGF-I deficiency, congenital GH deficiency, bone age, chronological age, puberty

## Abstract

**Highlights:**

**What are the main findings?**

- Bone age rather than chronological age is a useful index of pubertal maturation.
- In GH/IGF-I deficiency menarche occurs at a mean bone age of 14.

**What is the implication of the main finding?**

- Bone age is a useful tool to indicate pubertal stage.

**Abstract:**

**Background:** In the literature, the occurrence of menarche, the index of female sexual maturation, is related to chronological age (CA), but in conditions of abnormal growth it was proposed that determination of bone age (BA) was a better index of pubertal maturation than CA. **Aim:** To compare CA with BA at menarche in girls with Laron Syndrome (LS), a model of genetic IGF-I deficiency, which causes severe dwarfism and delayed puberty. **Subjects:** Data were retrieved retrospectively from our medical records. Nine untreated and four IGF-I-treated girls with LS were included in the study. Eleven GH-treated girls with congenital isolated GH deficiency (cIGHD) served as comparison. **Results**: The mean BA at menarche of untreated LS patients (13.4 y) was 0.7 y less than the CA (14.1 y), and the mean BA of IGF-I-treated girls (13.7 y) was 1.2 y less than the CA (14.9 y). Body height and weight did not influence the age at menarche. **Conclusion**: In girls with LS, the determination of BA seems a more useful index of female pubertal maturation than CA.

## 1. Introduction

Menarche is defined as the first menstrual period [1], which marks the peak process of biological maturation of puberty when girls become functionally capable of procreation [2]. Epidemiological studies conducted in several populations revealed a secular trend in advanced puberty, the chronological age at menarche decreasing in the last century [3,4]. This could be explained by changes in environmental factors such as better nutrition, attainment of optimal weight and height [5] and improved health by advances in medicine. On the other hand, delayed menarche may be caused by genetic factors [6], undernutrition [7] and intensive sporting activity [8]. Also, pituitary hormone deficiencies, such as growth hormone (GH) or IGF-I deficiency best exemplified by Laron Syndrome (LS) [9,10], lead to delayed puberty. Changes in growth and development are usually expressed using chronological age. Crampton first introduced the term “DEVELOPMENTAL AGE” [11]. It was subsequently demonstrated that this was better expressed by the estimation of the BONE (skeletal) AGE [BA] rather than by the CHRONOLOGICAL AGE (CA) [12].

Having found in a previous study in boys with LS that BA is a more precise index of pubertal maturation than CA at the time of *spermarche*, the male counterpart of *menarche* [13], the aim of the present study was to compare CA with BA at the time of *menarche* in girls with LS.

## 2. Subjects and Methods

This is a retrospective study. Data were retrieved from the medical records at the Endocrinology and Diabetes Research Unit, Schneider Children’s Medical Center. The criteria for the diagnosis of LS were: severe dwarfism, high serum growth hormone (GH), low or undetectable levels of serum IGF-I, unresponsive to GH stimulation, GH insensitivity and genetic analysis of the GH receptor gene [14]. Nine untreated and four IGF-I-treated girls with LS filled the criteria. Eleven girls with cIGHD treated with GH served as comparison. The diagnosis of cIGHD was made by clinical criteria, the failure of serum GH to rise above 4 µ/mL in response to insulin hypoglycemia and clonidine stimulation tests [15] and genetic analysis [16]. Menarche age was obtained by direct questioning of the patients and parents. Bone age was estimated by the same staff using the Atlas of Greulich and Pyle [17]. The statistical significance of the within-subject CA versus BA was calculated using Student’s paired analysis. The significance of the differences in means of growth parameters at menarche between the three groups was calculated by independent two-way Welch *t*-tests [18].

The study was approved by the Rabin Medical Center Ethics Committee (RMC-0611-24).

## 3. Results

The pertinent data at menarche for nine untreated and 4 IGF-I-treated girls with LS are shown in Table 1 and Table 2, and that for girls with cIGHD treated with GH in Table 3. The mean BA at menarche of untreated LS patients (13.4 y) was 0.7 y less than the CA (14.1 y), and the mean BA of IGF-I-treated LS girls (13.7 y) was 1.2 y less than the CA (14.9 y). Due to the small samples the differences were short of significance. The values of the mean BA and CA in girls with cIGHD were similar (13.4 y). Comparing the linear heights between the groups, it is evident that untreated girls with LS were much shorter at the time of menarche (mean ± SD 116.7 ± 9.6 cm) than the IGF-I-treated LS girls (130.6 ± 3.4 cm; *p* < 0.01) and the GH-treated girls with cIGHD (142.5 ± 5.4 cm; *p* < 0.001). Thus height did not affect the age at menarche. Also, the age of initiation of the hormone replacement or duration of therapy did not seem to influence the BA or CA at menarche. The mean weight at menarche of untreated LS girls was 26.9 ± 8.4 kg, denoting that a weight of over 48 kg is not needed for menarche to occur as proposed by Frisch and Revelle [5]. One girl with LS (Patient 4; Table 2) treated with IGF-I since the age of 3.7 years developed severe obesity of 66 kg. Her menarche occurred with a BA of 14 y at the CA of 15 years.

## 4. Discussion

Menarche is an important milestone in female sexual development [1] and a biological index of female sexual maturation [2]. The age of its occurrence is influenced by genetic, [6] nutritional [7,8] and hormonal factors. Among the latter is congenital IGF-I deficiency for which Laron Syndrome (LS) serves as a model [10]. Following a large cohort of patients with LS, our group had the opportunity to compare CA and BA as indexes of pubertal maturation. In a previous study in boys with LS we found that BA is a more reliable index of pubertal maturation, as expressed by age of spermarche, rather than CA [13]. The present study was aimed to determine whether this also holds true for the age at menarche in girls with LS. Indeed, we also found that in girls with LS, BA is a more consistent, less age-variable index of female maturation than CA. In an early study of pubertal development of 200 healthy girls, Simmons and Greulich [19] also found that chronological age is about twice as variable as skeletal age (BA) at the time of menarche. Our study also revealed that body height and duration or IGF-I or GH replacement treatment have no influence on the age at menarche. The finding that menarche occurs below a body weight of 30 kg contradicts the hypothesis of the need for a critical weight at menarche as proposed by Frisch and Revelle [5].

## 5. Conclusions

In untreated or IGF-I-treated girls with Laron Syndrome, the determination of bone age is a more useful index of pubertal maturation than chronological age.

## Figures and Tables

**Table 1:** Age and growth parameters at menarche of untreated girls with Laron Syndrome.

Patient No.	CA yr	BA yr	Ht cm	Ht SDS	Wt Kg
1	13.3	13.5	134.0	−3.6	40.5
2	14.7	13.0	107.0	−8.7	19.5
3	15.0	13.5	117.5	−7.3	
4	13.3	13.0	106.0	−7.9	17.5
5	14.5	13.4	127.0	−5.5	35.0
6	13.2	13.8	108.5	−7.1	18.5
7	13.2	14.6	118.5	−5.7	29.5
8	15.8	13.0	110.5	−8.6	23.5
9	14.0	12.5	121.2	−6.0	31.0
Mean	14.11	13.37	116.7	−6.7	26.9
SD	0.94	0.60	9.6	1.7	8.4
Range	13.2, 15.8	12.5, 14.6	107, 134	−8.7, −3.6	17.5, 40.5

CA—chronological age, BA—bone age, Ht—height, Ht SDS—Height Standard Deviation Score, Wt—weight.

**Table 2:** Age and growth parameters at menarche of girls with Laron Syndrome treated with IGF-I.

Patient	At Menarche					IGF-I Treatment	
No.	CA yr	BA yr	Ht cm	Ht SDS	Wt Kg	Start yr	Duration
1	15.7	13.1	130.5	−5.2	40.6	3.2	15.0
2	14.3	13.6	127.3	−5.4	36.9	3.5	13.1
3	14.8	14.5	134.0	−4.6	45.0	1.5	15.0
Mean	14.9	13.7	130.6	−5.1	40.8	2.7	14.4
SD	0.71	0.71	3.35	0.42	4.06	1.08	1.10
4	15	14	135	−4.4	66	3.7	12.3
Range	14.3, 15.7	13.1, 14.5	127.3, 134	−5.4, −4.6	36.9, 45.0	1.1, 3.5	13.1, 15.0

CA—chronological age, BA—bone age, Ht—height, Ht SDS—Height Standard Deviation Score, Wt—weight.

**Table 3:** Age and growth parameters at menarche of girls with cIGHD treated with GH.

Patient	At Menarche					GH Treatment	
No.	CA yr	BA yr	Ht cm	Ht SDS	Wt Kg	Start yr	Duration yr
1	14.8	13.5	134.6	−4.3	30.0	11.0	5.7
2	12.9	12.0	146.0	−1.4	40.0	7.5	6.5
3	14.0	13.6	144.0	−2.5	41.5	7.8	6.2
4	14.4	12.0	138.8	−4.0	32.7	12.5	3.7
5	11.8	12.0	134.0	−2.0	31.0	0.8	11.8
6	16.3	14.6	138.2	−4.0	37.0	9.0	8.6
7	14.5	15.0	147.4	−2.2	56.0	3.2	12.1
8	13.5	14.0	141.7	−2.5	50.5	5.5	9.3
9	12.4	12.6	147.0	−0.8	39.0	5.0	9.3
10	12.7	13.6	148.0	−0.8	49.5	1.1	12.0
11	14.5	14.0	148.0	−2.1	52.0	3.8	12.7
Mean	13.8	13.4	142.5	−2.4	41.7	6.1	8.9
SD	1.3	1.1	5.4	1.2	9.0	3.8	3.1
Range	11.8, 16.3	12.0, 15.0	134, 148	−4.3, −0.8	30, 56	0.8, 12.5	3.7, −12.7

CA—chronological age, BA—bone age, Ht—height, Ht SDS—Height Standard Deviation Score, Wt—weight.

## Data Availability

The data presented in this study are available on request from the corresponding author. The data are not publicly available due to restrictions privacy.
